# Editorial: Prognostication of Heart Failure Evolution: From Circulating Biomarkers to Genetic Risk Predictive Score

**DOI:** 10.3389/fcvm.2021.687232

**Published:** 2021-09-27

**Authors:** Alexander E. Berezin, Borut Peterlin, Michael Lichtenauer, Ioana Mozos

**Affiliations:** ^1^Internal Medicine Department, State Medical University, Ministry of Health of Ukraine, Zaporizhzhia, Ukraine; ^2^Clinical Institute of Medical Genetics, University Medical Center Ljubljana, Ljubljana, Slovenia; ^3^Division of Cardiology, Department of Internal Medicine II, Paracelsus Medical University Salzburg, Salzburg, Austria; ^4^Department of Functional Sciences–Pathophysiology, Center for Translational Research and Systems Medicine, “Victor Babes” University of Medicine and Pharmacy, Timisoara, Romania

**Keywords:** heart failure, biomarkers, stratification, prediction, risk scores

Circulating protein-based biomarkers are well-established tools for the diagnosis, stratification, and management of heart failure. Despite having a certain difference in biomarker panels that are recommended by the American Heart Association/American College of Cardiology and European Cardiology Society to routine clinical practice, cardiac biomarkers, mainly natriuretic peptides, demonstrate high discriminative ability to rule out the diagnosis of HF, and predict the risk of mortality/hospitalization among patients with overt HF. Although there are several challenges regarding the economic burden with biomarkers, their reproducibility, affordability, and interpretations of the results in serial measures, biomarker-based guided HF management, and prediction appear to be promising. However, there is no agreement in the design of various biomarker strategies (single vs. multiple biomarker panel, peak or serial measures, protein-based vs. transcriptomics-based biomarkers, and genetic scores vs. circulating biomarker scores), which could best fitted to each phenotype of HF and provide strong evidence for personified therapy of HF. We included four articles in the special issue “Prognostication of Heart Failure Evolution: From Circulating Biomarkers to Genetic Risk Predictive Score,” and these will be attractive for readers who are interesting in this topic.

Topf et al. reported a narrative review of 159 high-quality articles in the field of multiple biomarkers approaches to the management of HF with the aim to decrease mortality and improve quality of life. Authors provided in-depth analysis of the ability of several models consisted of biomarkers of the inflammatory axis, extracellular matrix remodeling, fibrosis, and oxidative stress to be a powerful diagnostic and therapeutic tool for patients having HF and found that multiple biomarker-based therapy has promising potential for providing tailored treatments among HF individuals.

The article provided by Berezin et al. depicts the challenging pathogenetic role of adipose tissue accumulation in the occurrence and development of HF among patients having several metabolic conditions. The wide spectrum of underlying molecular mechanisms, such as insulin resistance, oxidative stress, altered cardiac and vascular reparation, microvascular inflammation, and metabolic memory phenomenon, corresponded well with adverse cardiac remodeling, endothelial dysfunction, accelerating atherosclerosis, renal injury, and muscle weakness. Moreover, HF with preserved ejection fraction (HFpEF) rather than HF with reduced ejection fraction (HFrEF) can be concisely predicted through imbalance between pro- (adiponectin, leptin, resistin) and anti-inflammatory (visfatin, omentin, zinc-α2-glycoprotein, glypican-4) adipocytokines. Yet, dysregulation in natriuretic peptides and neprilysin synthesis occurred in obese patients and diabetics closely affecting adipocytokine signaling and local pro-inflammatory adipocytokine production may be a crucial mechanism, by which a switch of HF phenotypes from HFpEF to HFrEF becomes possible.

Shrivastava et al. concentrated on several circulating protein-based (brain-type natriuretic peptide, galectin 3, growth differentiation factor 15, heart-type fatty-acid-binding protein, mid-region of N-terminal prohormone of atrial-type natriuretic peptide, myosin binding protein-C; N-terminal prohormone of brain natriuretic peptide, and soluble suppression of tumorigenicity 2), genetic-based, and transcriptomics-based biomarkers, including non-coding RNAs, to develop polygenic risk scores for prediction of HF outcomes. The authors emphasized that genetic biomarkers did not significantly improve the discriminative value of classic cardiovascular risk factors and protein-based biomarkers. In addition, genetic risk scores appear to be time-consuming and expensive for clinical routine. Finally, a complementary panel constructed from combined circulating protein-based and transcriptomics-based biomarkers is a promising platform to improve individualized prediction and risk stratification of HF.

Zerbo et al. performed a systematic review of 24 articles to elucidate whether HF is the most important comorbidity that may be remarkably related to poor clinical outcomes and hematological alteration that occurred in cardiac implantable electronic device infection. Until recently, surgical interventions, such as cardiac implantable electronic device removal, and antimicrobial therapy in patients with infections had been considered as the gold standard. Investigators found that high pre-operative white blood cell count and elevated levels of C-reactive protein increased the risk of right ventricular failure development, and also increased red blood cell distribution width value or decreased platelet count was associated with poor clinical outcomes.

We believe that the topic of circulating biomarkers and multiple biomarker models offers a great opportunity for readers to find out more current challenges, benefits, and discoveries in the field of HF biomarkers. We hope that the special issue will open up for new prospective for investigations and attract new ideas for consideration in the future.

## Author Contributions

AB and ML have prepared the editorial with the consent from BP and IM. All authors contributed to the article and approved the submitted version.

## Conflict of Interest

The authors declare that the research was conducted in the absence of any commercial or financial relationships that could be construed as a potential conflict of interest.

## Publisher's Note

All claims expressed in this article are solely those of the authors and do not necessarily represent those of their affiliated organizations, or those of the publisher, the editors and the reviewers. Any product that may be evaluated in this article, or claim that may be made by its manufacturer, is not guaranteed or endorsed by the publisher.

